# Salt Equilibria and Protein Glycation in Young Child Formula

**DOI:** 10.3390/foods14193445

**Published:** 2025-10-08

**Authors:** Wenfu Chen, Wenzhu Yin, Xiumei Tao, Dasong Liu, Thom Huppertz, Xiaoming Liu, Peng Zhou

**Affiliations:** 1 School of Food Science and Technology, Jiangnan University, Wuxi 214122, Chinazhoupeng@jiangnan.edu.cn (P.Z.); 2 International Joint Research Laboratory for Dairy Science and Technology, Jiangnan University, Wuxi 214122, China; thom.huppertz@frieslandcampina.com; 3 School of Food and Nutritional Sciences, University College Cork, T12 YN60 Cork, Ireland; 4 FrieslandCampina, 3818 LE Amersfoort, The Netherlands

**Keywords:** young child formula, salt equilibria, calcium, phosphorus, casein mineralization, protein glycation

## Abstract

Young child formula (YCF) products are important sources of nutrients for children 1–3 years of age. Salt equilibria and protein glycation are two of the crucial aspects affecting nutritional properties and digestive behaviors of YCF, but detailed insights into these two aspects of YCF products remains limited. This study analyzed the distribution of salts and the level of protein glycation in 25 commercial YCF products from the retail market in China. The YCF products were reconstituted (12 g of powder per 100 g of water) and the distribution of calcium and phosphorus between the sedimentable (at 200× *g*), protein-associated and soluble (10 kDa-permeable) fractions were determined. Blocked lysine and 5-hydroxymethylfurfural were analyzed using reversed-phase high-performance liquid chromatography. Varying proportions of calcium (3.0–39.3%) and phosphorus (1.2–29.8%) were sedimentable for the products. Notable proportions of calcium (28.9–62.7%) and phosphorus (27.4–57.9%) were associated with the proteins. The remainder of the calcium (24.9–41.4%) and phosphorus (34.2–62.1%) were soluble. When expressing the protein-associated calcium as a function of casein, i.e., casein mineralization, large differences (~1.7 fold) were found among products. Variation in blocked lysine (7.4–19.2% of total lysine) and 5-hydroxymethylfurfural contents (3.0–7.0 mg/100 g protein) among products was also observed, suggesting notable differences in heat-load during processing. This study revealed notable variation in salt distribution and protein glycation among the YCF products. These findings underscore the critical need for manufacturers to optimize formulation and processing approaches, e.g., using milk with a low level of casein mineralization and using milk protein sources as concentrated liquid rather than powder to reduce protein glycation, to improve nutritional properties of the products.

## 1. Introduction

Young child formula (YCF) products are designed to be a complementary source of nutrients to the diet of young children of 1–3 years of age [1]. To accommodate their rapid growth and development, young children have increased nutritional requirements. Therefore, the provision of balanced and sufficient nutrients to fulfill the requirements of young children is the key purpose for YCF.

Besides the levels of salts, their distribution within the YCF products is also important [2]. In milk, the concentrations of calcium and phosphate are too high to be fully soluble, and therefore a partitioning of calcium and phosphate occurs [3,4]. One fraction of calcium and phosphate occurs as a soluble form in the serum fraction, and the other fraction is linked to the caseins, which is also referred to as the mineralization of caseins [2,5]. For cows’ milk, ~30% of calcium is soluble in the serum phase, whereas for human milk, ~65% of calcium is soluble in the serum phase [6,7]. The casein mineralization level is higher for bovine milk (~7.7 mM of associated Ca/10 g casein) than for breast milk (~3.2 mM of associated Ca/10 g casein) [6,8]. Furthermore, magnesium and citrate are also partially associated with the caseins in milk [9,10]. The mineralization of caseins is crucial for the formation of casein micelles, which are supramolecular assemblies of α_s_-, β- and κ-caseins and micellar calcium phosphate (MCP) [4]. The casein micelles in bovine milk (diameter ~150 nm) are larger than their human counterparts (~70 nm) [11]. Casein micelles are susceptible to calcium-mediated coagulation in the stomach following the hydrolysis of the κ-casein hairy layer by pepsin, which entails an important control over the transit of chyme [12,13]. Reducing the casein mineralization in a model infant formula led to no visible coagula and faster proteolysis of caseins under in vitro infant gastric conditions [14], whereas reducing the mineralization of casein micelles resulted in looser gastric coagula and faster casein digestion during an infant in vitro digestion [15]. In vivo studies in adults showed that ingesting milk with lower casein mineralization resulted in the gastric coagula emptying faster and the amino acids that are dominant in caseins (e.g., proline) concomitantly appearing faster in blood serum [16].

During the manufacturing of YCFs and their protein-containing ingredients, thermal treatment is typically applied in the thermal processing, concentration and drying processes [17,18]. Above a critical temperature, the whey proteins will undergo denaturation and form self-aggregates or complexes with caseins, which affect the solubility and digestibility of the proteins [19]. During the thermal treatment, the proteins are also susceptible to glycation, i.e., a reaction between the free amino groups of amino acid residues in proteins (e.g., side chain amino group of lysine) and the reactive carbonyl groups of reducing sugars (e.g., lactose) [20]. The initial stable products of the glycation reaction are Amadori compounds, such as lactulosyl-lysine, which can be quantified by determining furosine after thermal-acid hydrolysis [21,22]. 5-Hydroxymethylfurfural (HMF) is a typical intermediate stage Maillard reaction product derived from protein glycation, and is formed by the enolization of Amadori compounds [23,24]. The Amadori compounds render lysine biologically unavailable and also decrease the digestibility of the proteins by limiting protease accessibility to peptide bonds [19,25]. Increasing the protein glycation of an infant formula has been shown to result in a lower protein hydrolysis with the formation of larger peptides during infant in vitro gastrointestinal digestion [26]. In vivo, ingesting milk powder with a casein:whey protein ratio of 40:60 and with a higher level of protein glycation resulted in a lower postprandial plasma lysine concentration [27]. Furthermore, the intake of milk protein with a higher glycation attenuated postprandial plasma lysine availability for young males [28] and attenuated the postprandial increase in plasma lysine derived from the proteins [29].

Considering potential differences in formulation and processing approaches, it is hypothesized that salt distribution and protein glycation vary among YCF products, which in turn would affect their physicochemical and nutritional properties. Factors affecting salt equilibria and protein glycation and their effects on product properties have been extensively studied, especially for milk [8,30]. To date, there is still limited information regarding the salt distributions and protein glycation of YCF products, despite their importance in product quality. To bring insights into this field, the distribution of calcium, phosphorus, magnesium, potassium and sodium, and the protein-glycation-derived compounds including furosine and HMF in 25 commercial YCFs were analyzed.

## 2. Materials and Methods

### 2.1. Formulae Collection, Reconstitution and Fractionation

YCFs (n = 25) were obtained from commercial retailers in China. The sample set contained both products produced in China as well as products produced outside China (Table S1) and represented main brands of YCF in the Chinese retail market. For each product, three samples were taken, and each sample was measured once. A 12 g portion of each formula powder was reconstituted in 100 g of demineralized water and then stored overnight at 12 °C to achieve complete hydration.

According to the method previously described [2], formula dispersions were equilibrated at 25 °C for 1 h, and then centrifuged at 25 °C and 200× *g* for 30 min. The sediment and non-sedimentable phases were carefully separated via decanting. The non-sedimentable fraction was then centrifuged at 100,000× *g* for 1 h at 25 °C, after which a cream layer, a sediment and an intermittent layer were formed. The intermittent layer was collected using a syringe and then subjected to centrifugal ultrafiltration using a Vivaspin 6 concentrator (Sartorius Stedim Biotech GmbH, Goettingen, Germany) loaded with a 10 kDa membrane, after which the permeate was obtained. The whole sample, the non-sedimentable fraction and the 10 kDa permeate were used for salts analysis subsequently (see Section 2.3).

### 2.2. Formula Composition and pH Analysis

Total nitrogen (TN) and non-protein nitrogen (NPN) contents of the whole sample were measured by the Kjeldahl method, according to ISO/IDF (2014) [31] and ISO/IDF (2016) [32], respectively. To determine NPN, the whole sample was mixed (1:1, *v*/*v*) with 30% (*w*/*v*) trichloroacetic acid, followed by centrifugation at 10,000× *g* for 30 min. A 20 mL amount of the supernatant was used for the determination of nitrogen. Non-sedimentable (200× *g*) nitrogen content (NSN) was also determined using the Kjeldahl method. A nitrogen-to-protein conversion factor of 6.38 was used. A blank test using sucrose consumed 0.1 mL of titrant, and a recovery test using lysine hydrochloride and sucrose resulted in 99% recovery of nitrogen, indicating the validity of this method. The amounts of samples added for testing resulted in the consumption of 2.5–25 mL of titrant, which is above the detection limit.

The amino acid composition of the formulae was determined using an L-8900 Amino Acid Analyzer (Hitachi Ltd., Tokyo, Japan), according to the method previously described [33]. Casein as a percentage of total protein in the formulae was estimated from the concentrations of Phe, Pro, Asn+Asp and Ala, as previously described [34].

Fat content of the whole sample was determined using an alkaline hydrolysis method, as described by Chinese hygienic standard GB 5009.6-2016 [35]. The pH value of the whole sample was measured using a FE28 pH meter (Mettler Toledo Ltd., Shanghai, China).

### 2.3. Calcium, Phosphorus, Magnesium, Citrate, Potassium and Sodium Analysis

The whole sample, non-sedimentable fraction, and 10 kDa-permeable fraction were digested in nitric acid with a MARS Microwave Digestion apparatus (CEM Corp., Matthews, NC, USA) and then subject to determination using an iCAP TQ inductively coupled plasma mass spectrometer (Thermo Fisher Scientific, Inc., Bremen, Germany) for calcium, phosphorus, magnesium, potassium and sodium, respectively. The difference between the non-sedimentable and the 10 kDa-permeable fractions was assumed to be a protein-associated fraction.

For the determination of citrate content in the whole sample, the sample was mixed (1:1, *v*/*v*) with 30% (*w*/*v*) trichloroacetic acid, and then centrifuged at 10,000× *g* for 30 min. Citrate content of the supernatant was determined using an e2695 high-performance liquid chromatography (HPLC) unit (Waters Corp., Milford, MA, USA) loaded with a Waters Symmetry C18 column (250 × 4.6 mm I.D.), according to the method previously described [36,37].

### 2.4. Sodium Dodecyl Sulfate-Polyacrylamide Gel Electrophoresis (SDS-PAGE)

SDS-PAGE was performed under reducing and non-reducing conditions using a Mini-PROTEAN Tetra Cell apparatus (Bio-Rad Laboratories, Inc., Hercules, CA, USA) and gels a stacking gel (4%) and a separating gel (13%) [38]. The whole sample was diluted in ultrapure water to reach a protein content of 2 mg/mL, and then mixed (1:1, *v*/*v*) with sample buffers (62.5 mM of Tris-HCl, pH of 6.8, 2% (*w*/*v*) SDS, 25% (*v*/*v*) glycerol) with (reducing conditions) and without (non-reducing conditions) 5% (*v*/*v*) β-mercaptoethanol, respectively. After boiling for 3 min, 12 μL of the mixture was loaded in each well before electrophoresis. The gel was stained using 0.1% (*w*/*v*) Coomassie Brilliant Blue R-250, and then destained using 7.5% (*v*/*v*) acetic acid and 5% (*v*/*v*) methanol.

### 2.5. Furosine, Blocked Lysine, Reactive Lysine and HMF Analysis

For the determination of furosine content, 375 mg of formula powder was hydrolyzed in 8 mL of 6 M HCl at 110 °C for 22 h, and then diluted (1:5, *v*/*v*) with 6 g/L ammonium acetate, followed by filtration through a nylon membrane with a pore diameter of 0.22 μm (Fuji Science & Technology Co., Ltd., Tianjin, China). The furosine concentration of the filtrate was determined using the HPLC unit equipped with a Symmetry C18 column. The recovery of this method for formula samples added with furosine was 97% [39]. A quality control sample (4.2 mg/L furosine) was analyzed every 10 samples throughout the analytical sequences to ensure system stability. The determined furosine concentration was used together with the lysine concentration, determined in Section 2.2, to calculate the blocked and reactive lysine concentrations, according to the method previously described [40].

For HMF analysis, 2 g of formula powder was reconstituted in 10 g of demineralized water, and then 5 mL of 0.15 M oxalic acid was added. After heating at 95 °C for 25 min, 3 mL of 40% (*w*/*v*) trichloroacetic acid was added, followed by centrifugation at 6000× *g* for 15 min. The supernatant was filtered through the nylon membrane with a pore diameter of 0.22 μm. HMF concentration of the filtrate was determined using the HPLC unit loaded with the Symmetry C18 column, according to the method previously described [41]. The recovery of this method for formula samples added with HMF was 91% [41]. A quality control sample (4.0 mg/L HMF) was analyzed every 10 samples throughout the analytical sequences to ensure system stability.

## 3. Results

### 3.1. Formula Composition

**Figure 1:**
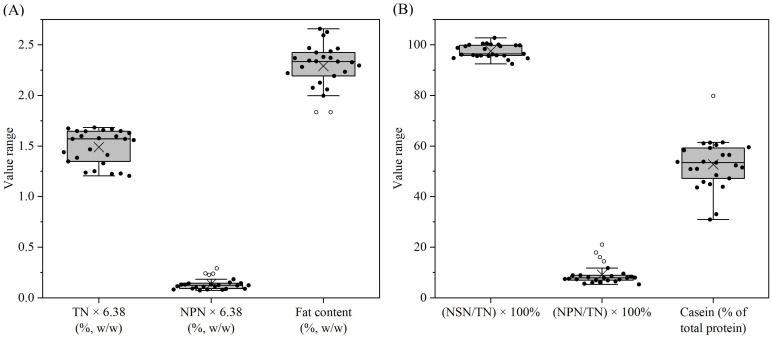
Total nitrogen (TN) × 6.38 (**A**), non-protein nitrogen (NPN) × 6.38 (**A**), fat content (**A**), non-sedimentable (200× *g*) nitrogen (NSN) as a percentage of TN (**B**), NPN as a percentage of TN (**B**), and casein as a percentage of total protein (**B**) for formulae dispersions (samples 1–25) prepared by reconstitution of 12 g of powder in 100 g of water. Data are shown in Box–Whisker plots. In the box, the x stands for the mean, the middle line stands for the median, and the bottom and the top lines stand for the lower and the upper quartiles, respectively. The whisker goes from the lowest to the highest values, leaving out the outliers whose distance from the upper or the lower quartile values surpasses 1.5× the interquartile distance. The inliers and outliers are shown as individual filled and hollow circles, respectively.

Crude protein contents of the reconstituted samples are shown in Figure 1A and Table S2. The protein contents of reconstituted samples varied from 1.21% to 1.68%, agreeing with the protein contents shown on product labels. After centrifugation at 200× *g* for 30 min, 92.4% of the total protein in sample 13 stayed in dispersion, and >95% of the total protein in most other samples stayed in dispersions (Figure 1B, Table S2). The NPN contents were <10% of total nitrogen for most samples. In four samples, NPN represented 14–21% of the total nitrogen (Figure 1B, Table S2). This is probably due to the addition of protein hydrolysates as an ingredient in these formulae. Free amino acids and peptides with ≤6 amino acids are soluble in 12% (*w*/*v*) trichloroacetic acid [42], which was used in the determination of NPN. Additionally, some highly glycosylated caseinomacropeptide (CMP) also remains soluble under this condition [43], and hence whey ingredients added in the formula would also contribute to an increase in the NPN content.

The percentages of casein in total protein for most samples were in the range of 44–61% (Figure 1B, Table S2). In sample 3, casein represented 80% of the total protein, whereas in samples 12 and 14, casein represented 31–33% of total protein, due to the addition of more whey protein ingredients in these formulae. With the growth of infants, the percentage of casein in the formula is adjusted to accommodate their nutritional requirements. For infant formula produced in China, the percentage of casein legally cannot be more than 40% [44]. For follow-on and young child formula produced in China, there is no specific regulation over the percentage of casein, and this is usually higher than that of infant formula, agreeing with the results of the present study (Table S2).

The fat contents of the reconstituted samples varied slightly, from 1.83% to 2.66% (Figure 1A, Table S2). Samples 7 and 8 had relatively lower fat contents, and samples 3, 14 and 23 had relatively higher fat contents. The determined fat contents were in line with the fat contents showing on the product labels.

### 3.2. pH Value

**Figure 2:**
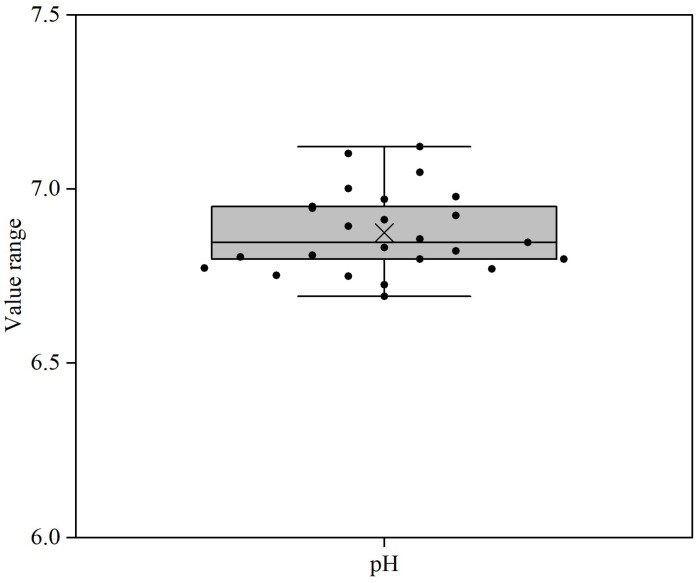
pH for formulae dispersions (samples 1–25) prepared by the reconstitution of 12 g of powder in 100 g of water. For an explanation of the Box–Whisker plot, see Figure 1.

pH values of the reconstituted samples are shown in Figure 2 and Table S3. The pH values of the samples varied from 6.7 to 7.2. Samples 11 and 22 showed a comparatively higher pH value, i.e., >7.0. The pH value of samples could be affected by the addition of probiotics, whey protein powders, salts, etc., in the formula, e.g., the addition of sodium or potassium citrate would result in an increase in the pH value.

### 3.3. Calcium Distribution

**Figure 3:**
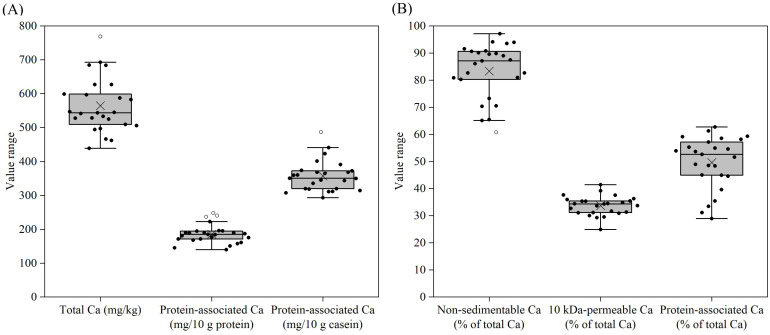
Total Ca (**A**), protein-associated Ca (mg/10 g protein) (**A**), protein-associated Ca (mg/10 g casein) (**A**), non-sedimentable (200× *g*) Ca as a percentage of total Ca (**B**), 10 kDa-permeable Ca as a percentage of total Ca (**B**), and protein-associated Ca as a percentage of total Ca (**B**) for formulae dispersions (samples 1–25) prepared by the reconstitution of 12 g of powder in 100 g of water. For an explanation of the Box–Whisker plots, see Figure 1.

The calcium contents of all the reconstituted samples ranged from 439 to 768 mg/kg (Figure 3A, Table S4). For most samples, <90% of calcium was non-sedimentable at 200× *g*; for samples 7, 8 and 24, the non-sedimentable calcium represented only 60–65% of the total calcium (Figure 3B, Table S4). The average percentage of non-sedimentable calcium for all samples was 83% of the total calcium. Given that most protein was non-sedimentable for these samples (Figure 1B), the sedimentable calcium likely primarily arises from the addition of sparingly soluble calcium salts such as calcium carbonate or calcium phosphates to the formulae. Across the samples, 25–41% of calcium was 10 kDa-permeable and 29–63% of calcium was associated with the proteins (Figure 3B, Table S4). The protein-associated calcium represented a major calcium fraction in most samples. When the protein-associated calcium was expressed as a function of protein instead of total calcium, different trends with smaller variations among samples were observed (Figure 3A, Table S4). When the protein-associated calcium was shown as a function of casein, i.e., as a level of casein mineralization, much more different trends with much smaller variations among samples were observed (Figure 3A, Table S4). The average mineralization level for all samples was 357 mg of calcium/10 g of casein, with samples 21 and 14 showing the lowest and highest values of 293 and 487 mg of calcium/10 g of casein, respectively. These differences might be attributed to the formulation choices for these formulae.

### 3.4. Phosphorus Distribution

**Figure 4:**
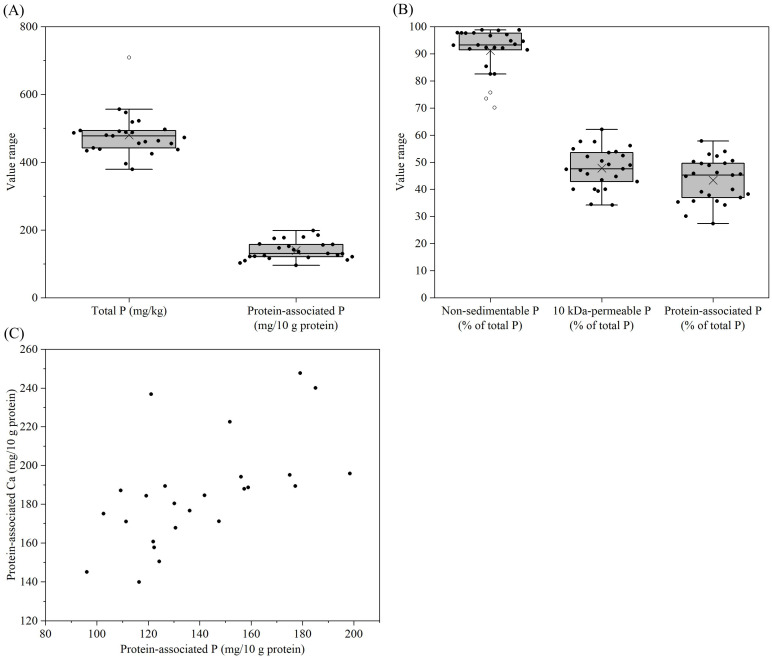
Total P (**A**), protein-associated P (mg/10 g protein) (**A**), non-sedimentable (200× *g*) P as a percentage of total P (B), 10 kDa-permeable P as a percentage of total P (**B**), protein-associated P as a percentage of total P (**B**), and the correlation between protein-associated P and Ca (**C**) for formulae dispersions (samples 1–25) prepared by the reconstitution of 12 g of powder in 100 g of water. For an explanation of the Box–Whisker plots, see Figure 1.

The phosphorus content of all the reconstituted samples was in the range of 379–709 mg/kg, with the highest value found in sample 4 (Figure 4A, Table S5). For most samples, >90% of phosphorus was non-sedimentable at 200× *g*, but for samples 4, 13 and 24, only 70–76% of phosphorus was non-sedimentable (Figure 4B, Table S5). The sedimentable phosphorus was primarily regarded as phosphorus in the form of sparingly soluble phosphate salts such as calcium phosphates. Similarly to calcium, a low percentage of phosphorus was 10 kDa-permeable in all samples, i.e., only 34–62% of phosphorus was permeable (Figure 4B, Table S5). In all samples, 27–58% of phosphorus was protein-associated, either in the form of phosphate groups esterified to serine residues or inorganic phosphate as part of MCP (Figure 4B, Table S5). The protein-associated phosphorus represented a notable fraction in all samples. When the protein-associated phosphorus was expressed as a function of protein instead of total phosphorus, different trends with larger variations among samples were observed (Figure 4A, Table S5). This suggested that other factors such as the exogenously added phosphate salts might have notable effects on the level of protein-associated phosphorus. For all samples, the protein-associated phosphorus contents showed a generally positive correlation with the protein-associated calcium contents (Figure 4C), indicating their co-existence in the form of MCP in casein micelles.

### 3.5. Distribution of Other Salts

**Figure 5:**
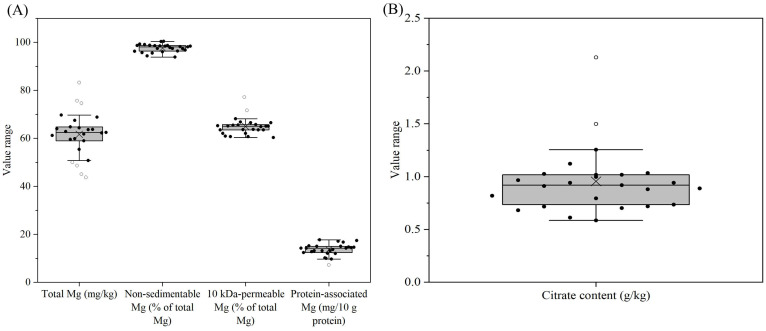
Total Mg (**A**), non-sedimentable (200× *g*) Mg as a percentage of total Mg (**A**), 10 kDa-permeable Mg as a percentage of total Mg (**A**), protein-associated Mg (mg/10 g protein) (**A**), and citrate content (**B**) for formulae dispersions (samples 1–25) prepared by the reconstitution of 12 g of powder in 100 g of water. For an explanation of the Box–Whisker plots, see Figure 1.

The magnesium contents of all the reconstituted samples were in the range of 44–83 mg/kg (Figure 5A, Table S6). In almost all samples, >95% of magnesium was non-sedimentable at 200× g. Similarly to calcium and phosphorus, low percentages of magnesium were 10 kDa-permeable for all samples, i.e., only 60–77% of magnesium was permeable (Figure 5A, Table S6). The 10 kDa-impermeable magnesium were mostly linked to the proteins. Citrate was also determined for the reconstituted samples. The citrate contents of all samples varied from 0.59 to 2.13 g/kg, with the lowest and highest values found for samples 10 and 23, respectively (Figure 5B, Table S6). Citrate could be added to the formula in the form of potassium/sodium citrate or calcium citrate, which would differently affect the calcium distribution of the samples.

**Figure 6:**
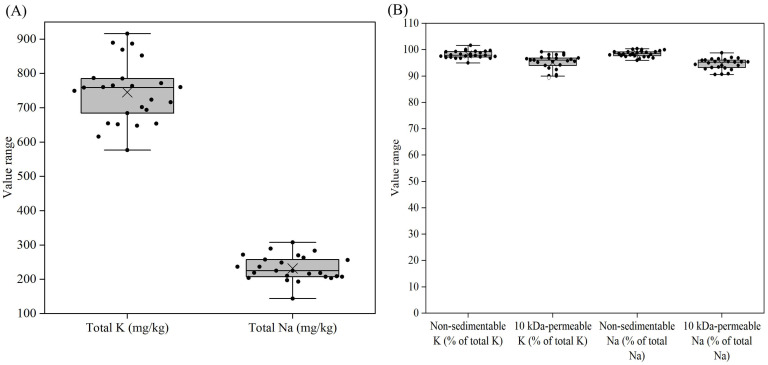
Total K (**A**), total Na (**A**), non-sedimentable (200× *g*) K as a percentage of total K (**B**), 10 kDa-permeable K as a percentage of total K (**B**), non-sedimentable (200× *g*) Na as a percentage of total Na (**B**), and 10 kDa-permeable Na as a percentage of total Na (**B**) for formulae dispersions (samples 1–25) prepared by the reconstitution of 12 g of powder in 100 g of water. For an explanation of the Box–Whisker plots, see Figure 1.

The potassium contents of all the reconstituted samples ranged from 577 to 916 mg/kg, with the lowest and highest values found in samples 3 and 21, respectively (Figure 6A, Table S7). Unlike calcium and phosphorus, in all samples, more than 95% of potassium was found to be non-sedimentable after 30 min of centrifugation at 200× *g*, and more than 90% of potassium was permeable through a 10 kDa membrane (Figure 6B, Table S7). Sodium showed a similar trend. The sodium contents of all the reconstituted samples ranged from 144 to 308 mg/kg, with >95% of sodium being non-sedimentable and >90% of sodium being 10 kDa-permeable (Figure 6B, Table S7). The fractions of potassium and sodium that were not 10 kDa-permeable, were likely associated with proteins as counterions for negatively charged amino acid residues.

### 3.6. SDS-PAGE Patterns

**Figure 7:**
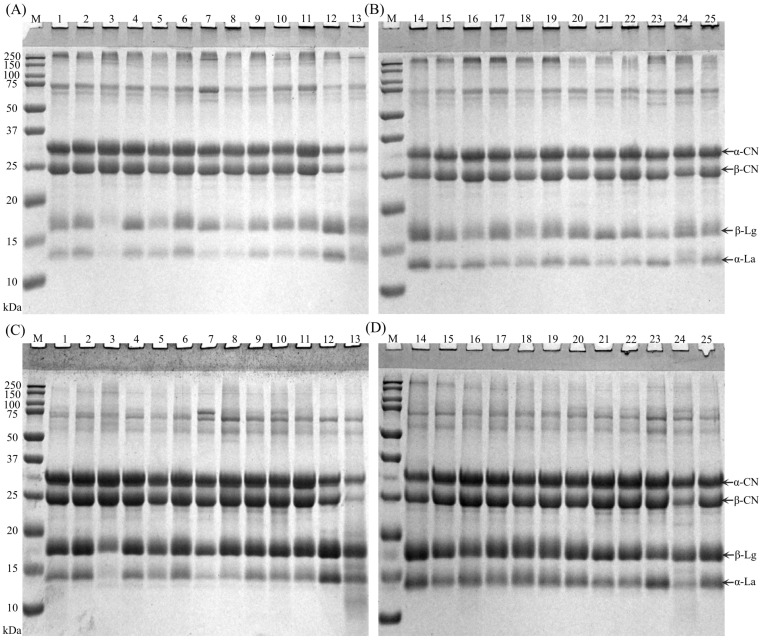
Non-reducing (**A**,**B**) and reducing (**C**,**D**) SDS-PAGE patterns for formulae dispersions of samples 1–25 prepared by the reconstitution of 12 g of powder in 100 g of water. M, protein markers; CN, casein; Lg, lactoglobulin; La, lactalbumin.

For all samples, the non-reducing SDS-PAGE patterns showed protein aggregates in the stacking gel and also in the upper part of the resolving gel corresponding to a molecular weight of ~250 kDa (Figure 7A,B); these were not observed for the reducing SDS-PAGE patterns (Figure 7C,D). This suggests the occurrence of intermolecular disulfide bonding that contributed to the aggregation of proteins. For the non-reducing SDS-PAGE patterns, with the disappearance of large protein aggregates, the band intensity for β-lactoglobulin and α-lactalbumin increased notably, especially for samples 3, 5 and 8, suggesting the involvement of the two major whey proteins in forming the aggregates. The presence of protein aggregates might reflect the magnitude of thermal treatment encountered during the manufacturing of the formulae and their protein ingredients. For sample 3, the band intensity for β-lactoglobulin and α-lactalbumin was notably weaker compared to caseins; while for samples 12 and 14, the band intensity for caseins was weaker compared to β-lactoglobulin and α-lactalbumin, agreeing with the percentage of casein in total protein (Table S2). For sample 13, a notably diffuse background was observed, agreeing with the content of NPN (Table S2).

### 3.7. Furosine, Blocked Lysine, Reactive Lysine and HMF Contents

**Figure 8:**
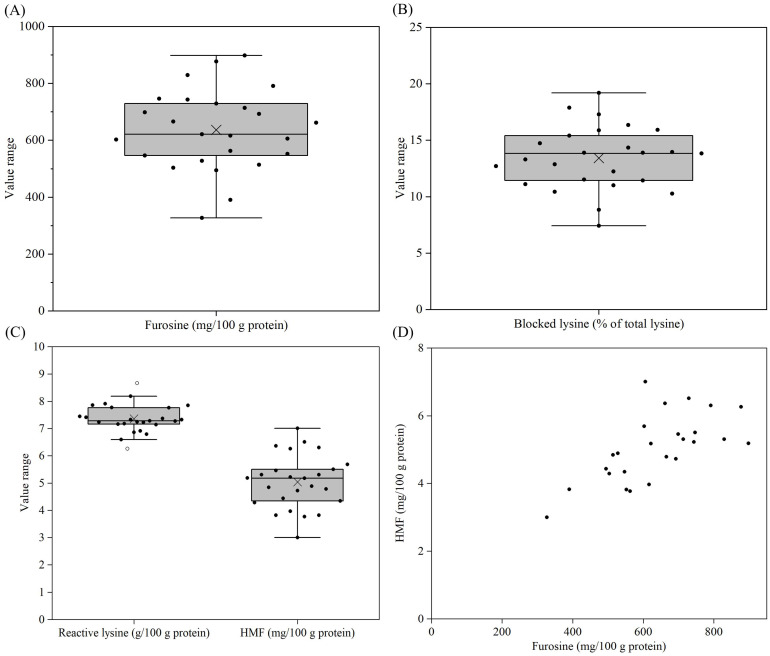
Furosine content (**A**), blocked lysine as a percentage of total lysine (**B**), reactive lysine content (**C**), HMF content (**C**), and the correlation between furosine and HMF content (**D**) for formulae dispersions (samples 1–25) prepared by the reconstitution of 12 g of powder in 100 g of water. For an explanation of the Box–Whisker plots, see Figure 1.

The furosine content of the samples varied between 327 and 899 mg/100 g protein, with the lowest and highest values found for samples 21 and 25, respectively (Figure 8A, Table S8). Furosine is released during the thermal-acid hydrolysis of proteins containing blocked lysine as lactulosyl-lysine [21]. When converting furosine contents into percentages of blocked lysine in total lysine, a similar trend with large variations among samples was also observed (Figure 8B, Table S8). The percentage of blocked lysine ranged from 7.4 to 19.2% of total lysine. For reactive lysine contents, a different trend with smaller variation among samples were observed, compared to blocked lysine. The reactive lysine contents of all samples were in the range of 6.3 to 8.7 g/100 g protein, with the highest value found for sample 14 (Figure 8C, Table S8). Considering that the total lysine contents vary among different proteins, the percentage of blocked lysine could be more closely related to the thermal history of the infant formulae and their protein ingredients.

HMF was also determined for the reconstituted samples (Figure 8C, Table S8). Like furosine, the HMF contents also showed larger variations among samples, but with a different trend. The HMF contents for all samples were in the range of 3.0–7.0 mg/100 g protein, with the lowest value found for sample 21. For all samples, the HMF contents showed a generally positive correlation with the furosine contents (Figure 8D), indicating a further progression of the initial glycation reaction for all products.

## 4. Discussion

The YCF products showed notable variation in salt distribution among sedimentable (at 200× *g*), protein-associated and soluble (10 kDa-permeable) fractions. Very little magnesium in the samples was sedimentable (Figure 5A, Table S6), while more notable levels of calcium and phosphorus were sedimentable (Figure 3B and Figure 4B), probably due to the addition of sparingly soluble salts, such as calcium carbonate or calcium phosphate, to some of the YCF products. A large portion of non-sedimentable magnesium, calcium and phosphorus in samples was associated with the proteins (Figure 3B, Figure 4B and Figure 5A). In bovine milk, ~50% of magnesium, ~70% of calcium and ~50% of phosphate are also associated with casein micelles since the solubilities of the respective salts are less than their concentrations [45]. Therefore, the addition of liquid or powdered milk as an ingredient contributes the protein-associated calcium into the formulae. The majority of protein-associated calcium is associated with the caseins, and hence the level of protein-associated calcium essentially reflects the level of casein mineralization of the products [2]. The majority of potassium and sodium in all samples were soluble (Figure 6B, Table S7), and the other fractions were probably associated with proteins as counterions for the amino acid residues with negative charges.

The level of casein mineralization varied notably among the YCF products (Figure 3A, Table S4). Any factors affecting the solubility of calcium phosphate will in turn affect the mineralization of casein micelles in the products. A lower pH leads to an increase in the solubility of calcium phosphate [30], but the small variation in pH (6.7–7.2) between samples alone cannot account for the large variation in casein mineralization (Figure 2, Table S3). The addition of salts directly or through whey protein ingredients to the formulae can affect casein mineralization. The addition of soluble calcium salts (e.g., CaCl_2_) or soluble phosphate salts (e.g., Na_2_HPO_4_) to the formulae increases the supersaturation of calcium phosphate, and therewith casein mineralization [46], whereas the addition of citrate salts reduces casein mineralization due to the chelation of calcium [47]. Additionally, the variation in casein mineralization in products can also be attributed to sources of milk, which showed notable differences in casein mineralization among breeds [48] and even among individual cows of the same breed [49].

Milk with lower levels of casein mineralization has been shown to have poorer rennet-induced coagulation properties [3]. A clinical trial also showed that reducing casein mineralization of milk by adding trisodium citrate resulted in a faster empty of gastric coagula and an earlier appearance of the amino acids (e.g., proline) that are dominant in caseins in serum [16]. Although carried out in adults rather than young children or infants, the study provided valuable insights into the effect of casein mineralization on coagulation behaviors in vivo. This was consistent with our previous in vitro study, which showed that reducing the mineralization level of casein micelles by ion-exchange resulted in the formation of looser coagula that promoted casein degradation during in vitro gastric digestion under infant, adult and elderly conditions, respectively, due probably to a higher accessibility of digestive proteases to peptide bonds [15]. The formation of dense gastric coagula is associated with the issues of reflux esophagitis and difficulties in defecation in infants [50]. The lower levels of casein mineralization together with higher levels of non-micellar caseins could weaken the coagulation of para-casein after the hydrolysis of κ-casein hairy layer by pepsin [11]. For young children of 1–3 years of age, the gastrointestinal physiology is quite mature, e.g., secretion of pepsin, gastric acid and pancreatic proteases reaches adult levels at 2 years of age, 6 months and 1 month, respectively [51]. Hence, insights from in vivo studies in adults also provide valuable insights for young children. Both in vitro studies under different age conditions and in vivo studies in adults showed an overall weakened gastric coagulation for casein micelles with lower levels of mineralization. An in vivo study in young children is warranted to validate the influence of casein mineralization on gastric coagulation, amino acid absorption and physiological outcomes such as growth and development. Magnesium shows a low content in YCF products, and potassium and sodium are monovalent ions, thus having limited effects on gastric coagulation of casein micelles. These salts would affect the osmotic pressure of the products and should also be considered.

The level of protein glycation also varied notably among the YCF products. This is related to the thermal history of the products and their protein-containing ingredients. To change the casein:whey protein ratio in the products, the whey protein-containing ingredient is typically mixed with milk before further processing. If the whey protein is added as a powder to the milk, two times of drying processes will be encountered, with one for the whey protein powder and the other for the formulae powder. This could result in a higher level of whey protein glycation in the products, compared to the addition of whey protein as concentrated liquid [52]. Similarly, milk powder rather than fresh milk is often used during the wet-mix process, which could be another source of more protein glycation. Additionally, the accumulative thermal load during the heat treatment, concentration and spray-drying processes for the manufacturing of YCF also affects the level of protein glycation [53]. The glycation reaction causes a decrease in nutritional value through blocking the essential amino acid lysine and through decreasing the digestibility of the proteins [54]. Several clinical trials, in young males, showed that the glycation of milk proteins resulted in attenuated postprandial plasma lysine availability [28,29]. The glycation of proteins could also result in the formation of potential toxic compounds such as HMF and dicarbonyl compounds, which could induce cytotoxic issues in cells and animals, such as decreasing antioxidant-glutathione [55].

**Figure 9:**
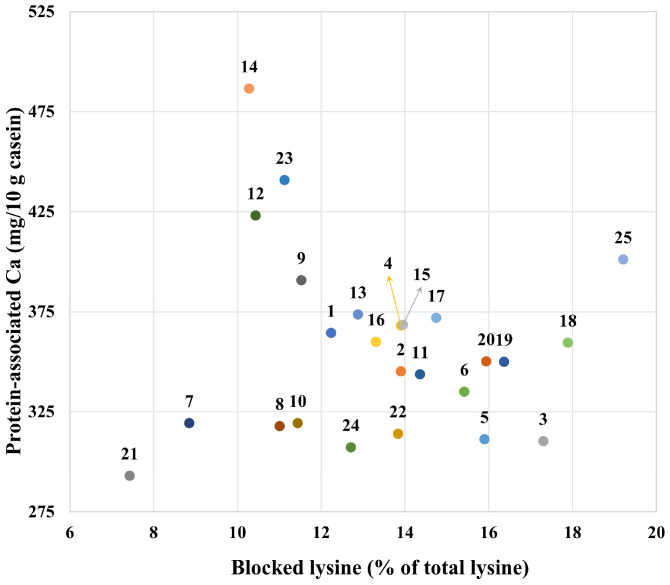
Plot of protein-associated calcium vs. blocked lysine as a percentage of total lysine for formulae dispersions (samples 1–25) prepared by the reconstitution of 12 g of powder in 100 g of water.

A plot of protein-associated calcium vs. blocked lysine showed large variation among the YCF products (Figure 9). The amount of protein associated calcium varied 1.7-fold, and the blocked lysine as a percentage of total lysine varied 2.6-fold. Sample 21 showed the minimum values for both the protein-associated calcium and the blocked lysine, which, as outlined above, is considered beneficial. Sample 14 showed the highest value for the protein-associated calcium, and sample 25 showed the maximum value for the blocked lysine. These results suggest a critical need for manufacturers to optimize formulation and processing approaches to reduce both the levels of casein mineralization and protein glycation, e.g., by increasing the ratio of citrate in salts and by using whey protein as a concentrated liquid rather than powder. This would improve the physicochemical and nutritional properties of the YCF products. With the aid of this plot, typical products could be carefully selected to check the effects of casein mineralization and protein glycation on their digestibility and absorption.

## 5. Conclusions

This study highlighted notable variations in salt distribution and protein glycation among the YCF products. The protein-associated calcium and phosphorus are of crucial importance, as they are directly associated with the mineralization of casein micelles. The levels of casein micelle mineralization and protein glycation could have a potentially significant effect on the digestibility and nutrient provision of the products. Therefore, the distribution of salts (i.e., not only concentration) and the thermal treatment to both products and ingredients should be carefully considered when manufacturing YCF. To reduce the level of casein mineralization, milk with a low level of casein mineralization could be used, and the ratio of citrate in salts could be increased. To reduce the level of protein glycation, fresh milk rather than milk powder as well as whey protein as a concentrated liquid rather than a powder could be used.

## Data Availability

All the data are available from the first author upon reasonable request.

## Supplementary Materials

The following supporting information can be downloaded at: https://www.mdpi.com/article/10.3390/foods14193445/s1, Table S1: Producers and countries of production for samples 1–25. Different upper-case letters stand for different producers, and a same upper-case letter followed by different numbers stand for different products from a same producer; Table S2: Total nitrogen (TN) × 6.38, non-protein nitrogen (NPN) × 6.38, fat content, non-sedimentable (200× *g*) nitrogen (NSN) as a percentage of TN, NPN as a percentage of TN, and casein as a percentage of total protein for formulae dispersions (samples 1–25) prepared by reconstitution of 12 g powder in 100 g water; Table S3: pH for formulae dispersions (samples 1–25) prepared by reconstitution of 12 g powder in 100 g water; Table S4: Total Ca, protein-associated Ca (mg/10 g protein), protein-associated Ca (mg/10 g casein), non-sedimentable (200× *g*) Ca as a percentage of total Ca, 10 kDa-permeable Ca as a percentage of total Ca, and protein-associated Ca as a percentage of total Ca for formulae dispersions (samples 1–25) prepared by reconstitution of 12 g powder in 100 g water; Table S5: Total P, protein-associated P (mg/10 g protein), non-sedimentable (200× *g*) P as a percentage of total P, 10 kDa-permeable P as a percentage of total P, and protein-associated P as a percentage of total P for formulae dispersions (samples 1–25) prepared by reconstitution of 12 g powder in 100 g water; Table S6: Total Mg, non-sedimentable (200× *g*) Mg as a percentage of total Mg, 10 kDa-permeable Mg as a percentage of total Mg, protein-associated Mg (mg/10 g protein), and citrate content for formulae dispersions (samples 1–25) prepared by reconstitution of 12 g powder in 100 g water; Table S7: Total K, total Na, non-sedimentable (200× *g*) K as a percentage of total K, 10 kDa-permeable K as a percentage of total K, non-sedimentable (200× *g*) Na as a percentage of total Na, and 10 kDa-permeable Na as a percentage of total Na for formulae dispersions (samples 1–25) prepared by reconstitution of 12 g powder in 100 g water; Table S8: Furosine content, blocked lysine as a percentage of total lysine, reactive lysine content, and HMF content for formulae dispersions (samples 1–25) prepared by reconstitution of 12 g powder in 100 g water.
