# Supplementary material for: Salt Equilibria and Protein Glycation in Young Child Formula
Source: Foods. 2025 Oct 8;14(19):3445. doi: 10.3390/foods14193445 (PMC12523973; doi:10.3390/foods14193445)
Supplement: Supplementary file 1 [file foods-14-03445-s001.zip › foods-3854602-supplementary.docx]

**Table S1.** Producers and countries of production for samples 1-25. Different upper-case letters stand for different producers, and a same upper-case letter followed by different numbers stand for different products from a same producer.

| Sample | Producer | Product | Country of production |
| --- | --- | --- | --- |
| 1 | A | A1 | China |
| 2 | A | A2 | China |
| 3 | B | B1 | Netherlands |
| 4 | B | B2 | Netherlands |
| 5 | C | C1 | China |
| 6 | C | C2 | China |
| 7 | D | D1 | Netherlands |
| 8 | D | D2 | Netherlands |
| 9 | E | E1 | France |
| 10 | E | E2 | France |
| 11 | F | F1 | New Zealand |
| 12 | G | G1 | France |
| 13 | H | H1 | Netherlands |
| 14 | I | I1 | China |
| 15 | J | J1 | China |
| 16 | K | K1 | China |
| 17 | K | K2 | China |
| 18 | K | K3 | China |
| 19 | K | K4 | China |
| 20 | L | L1 | China |
| 21 | M | M1 | Netherlands |
| 22 | N | N1 | Netherlands |
| 23 | O | O1 | Switzerland |
| 24 | P | P1 | Netherlands |
| 25 | Q | Q1 | China |

**Table S2.** Total nitrogen (TN) × 6.38, non-protein nitrogen (NPN) × 6.38, fat content, non-sedimentable (200 × *g*) nitrogen (NSN) as a percentage of TN, NPN as a percentage of TN, and casein as a percentage of total protein for formulae dispersions (samples 1-25) prepared by reconstitution of 12 g powder in 100 g water.

| Sample | TN × 6.38  (%, w/w) | NPN × 6.38  (%, w/w) | Fat content  (%, w/w) | (NSN/TN) × 100% | (NPN/TN) × 100% | Casein (% of total protein) |
| --- | --- | --- | --- | --- | --- | --- |
| 1 | 1.66±0.02 | 0.239±0.006 | 2.06±0.02 | 96.2±0.0 | 14.4±0.4 | 48.5±0.4 |
| 2 | 1.68±0.01 | 0.133±0.003 | 2.12±0.01 | 95.6±0.3 | 7.9±0.2 | 53.5±0.6 |
| 3 | 1.33±0.00 | 0.082±0.006 | 2.59±0.02 | 100.1±0.3 | 6.2±0.4 | 79.8±0.8 |
| 4 | 1.67±0.00 | 0.115±0.002 | 2.00±0.02 | 95.9±0.2 | 6.9±0.1 | 60.4±0.5 |
| 5 | 1.58±0.01 | 0.110±0.002 | 2.38±0.02 | 98.3±0.0 | 7.0±0.1 | 59.2±0.2 |
| 6 | 1.47±0.01 | 0.126±0.007 | 2.34±0.01 | 99.4±0.6 | 8.6±0.5 | 56.5±0.6 |
| 7 | 1.25±0.02 | 0.074±0.002 | 1.83±0.02 | 95.7±0.1 | 5.9±0.2 | 61.3±0.5 |
| 8 | 1.22±0.00 | 0.080±0.002 | 1.83±0.02 | 95.8±0.1f | 6.5±0.2 | 53.8±0.2 |
| 9 | 1.24±0.01 | 0.100±0.001 | 2.37±0.02 | 95.6±1.1 | 8.1±0.0 | 61.4±1.1 |
| 10 | 1.23±0.01 | 0.089±0.003 | 2.34±0.02 | 93.9±0.1 | 7.2±0.2 | 61.1±0.9 |
| 11 | 1.65±0.01 | 0.092±0.003 | 2.42±0.02 | 96.0±0.4 | 5.6±0.2 | 56.5±1.3 |
| 12 | 1.41±0.01 | 0.226±0.003 | 2.34±0.02 | 100.5±0.8 | 16.0±0.2 | 33.1±0.5 |
| 13 | 1.38±0.01 | 0.291±0.003 | 2.43±0.01 | 92.4±1.2 | 21.0±0.2 | 44.9±0.7 |
| 14 | 1.60±0.00 | 0.153±0.001 | 2.63±0.02 | 102.8±1.2 | 9.5±0.1 | 30.9±1.3 |
| 15 | 1.59±0.00 | 0.142±0.004 | 2.19±0.02 | 100.5±0.5 | 8.9±0.2 | 51.0±1.3 |
| 16 | 1.57±0.02 | 0.185±0.003 | 2.28±0.03 | 100.0±1.0 | 11.8±0.2 | 52.4±0.4 |
| 17 | 1.65±0.00 | 0.130±0.002 | 2.23±0.02 | 99.8±0.5 | 7.9±0.1 | 50.9±0.4 |
| 18 | 1.65±0.00 | 0.121±0.002 | 2.37±0.01 | 99.5±0.1 | 7.3±0.1 | 43.9±1.2 |
| 19 | 1.57±0.01 | 0.131±0.001 | 2.07±0.02 | 99.8±0.2 | 8.3±0.1 | 51.5±0.8 |
| 20 | 1.63±0.01 | 0.145±0.002 | 2.33±0.01 | 100.1±0.6 | 8.9±0.1 | 45.9±1.1 |
| 21 | 1.44±0.00 | 0.115±0.005 | 2.22±0.02 | 96.2±0.5 | 8.0±0.4 | 58.3±0.3 |
| 22 | 1.21±0.01 | 0.091±0.002 | 2.47±0.02 | 96.4±0.2 | 7.5±0.2 | 59.5±1.4 |
| 23 | 1.56±0.02 | 0.082±0.004 | 2.66±0.03 | 98.8±0.2 | 5.3±0.3 | 53.7±0.4 |
| 24 | 1.35±0.00 | 0.242±0.003 | 2.46±0.02 | 94.7±0.1 | 17.9±0.2 | 47.2±0.7 |
| 25 | 1.67±0.02 | 0.124±0.005 | 2.30±0.02 | 94.8±0.1 | 7.4±0.3 | 43.6±0.1 |

**Table S3.** pH for formulae dispersions (samples 1-25) prepared by reconstitution of 12 g powder in 100 g water.

| Sample | pH |
| --- | --- |
| 1 | 6.86±0.00 |
| 2 | 6.89±0.01 |
| 3 | 6.97±0.01 |
| 4 | 6.83±0.00 |
| 5 | 6.81±0.01 |
| 6 | 6.82±0.00 |
| 7 | 6.95±0.01 |
| 8 | 7.05±0.00 |
| 9 | 6.91±0.00 |
| 10 | 6.80±0.00 |
| 11 | 7.12±0.01 |
| 12 | 6.75±0.01 |
| 13 | 6.73±0.00 |
| 14 | 6.77±0.01 |
| 15 | 6.75±0.00 |
| 16 | 6.98±0.00 |
| 17 | 6.80±0.00 |
| 18 | 7.00±0.00 |
| 19 | 6.95±0.01 |
| 20 | 6.69±0.00 |
| 21 | 6.81±0.00 |
| 22 | 7.10±0.01 |
| 23 | 6.77±0.00 |
| 24 | 6.92±0.00 |
| 25 | 6.85±0.01 |

**Table S4.** Total Ca, protein-associated Ca (mg/10 g protein), protein-associated Ca (mg/10 g casein), non-sedimentable (200 × *g*) Ca as a percentage of total Ca, 10 kDa-permeable Ca as a percentage of total Ca, and protein-associated Ca as a percentage of total Ca for formulae dispersions (samples 1-25) prepared by reconstitution of 12 g powder in 100 g water.

| Sample | Total Ca (mg/kg) | Protein-associated Ca (mg/10 g protein) | Protein-associated Ca (mg/10 g casein) | Non-sedimentable Ca (% of total Ca) | 10 kDa-permeable Ca (% of total Ca) | Protein-associated Ca (% of total Ca) |
| --- | --- | --- | --- | --- | --- | --- |
| 1 | 533.4±1.0 | 176.6±2.4 | 364.4±4.9 | 94.1±0.8 | 39.2±0.3 | 54.9±0.7 |
| 2 | 543.2±1.0 | 184.5±1.2 | 345.2±2.3 | 90.8±0.6 | 33.6±0.3 | 57.1±0.4 |
| 3 | 524.9±1.6 | 247.7±2.8 | 310.4±3.5 | 97.1±0.3 | 34.3±0.6 | 62.7±0.7 |
| 4 | 768.3±4.0 | 222.5±4.1 | 368.2±6.8 | 73.2±0.2 | 24.9±0.7 | 48.3±0.9 |
| 5 | 497.0±8.6 | 184.3±3.5 | 311.3±5.8 | 89.6±1.2 | 31.1±0.6 | 58.5±1.1 |
| 6 | 528.2±3.8 | 189.4±5.1 | 335.1±9.0 | 87.1±0.8 | 34.5±0.7 | 52.6±1.4 |
| 7 | 692.5±5.5 | 195.9±5.4 | 319.4±8.8 | 65.4±0.9 | 30.0±0.9 | 35.4±1.0 |
| 8 | 627.0±18.4 | 171.1±1.8 | 317.9±3.3 | 65.1±0.3 | 31.7±0.3 | 33.4±0.3 |
| 9 | 544.8±7.1 | 240.1±2.4 | 390.8±3.9 | 89.9±0.1 | 35.3±0.5 | 54.5±0.5 |
| 10 | 493.8±14.0 | 195.1±5.1 | 319.4±8.4 | 86.0±1.2 | 37.5±0.1 | 48.5±1.3 |
| 11 | 596.5±2.2 | 194.1±2.7 | 343.7±4.8 | 88.9±0.2 | 35.3±0.6 | 53.6±0.7 |
| 12 | 683.5±9.8 | 139.9±3.2 | 423.2±9.6 | 70.3±0.2 | 41.4±0.6 | 28.9±0.7 |
| 13 | 587.2±7.6 | 167.8±1.9 | 373.7±4.3 | 70.5±0.5 | 30.9±0.7 | 39.6±0.5 |
| 14 | 466.1±3.3 | 150.5±2.9 | 486.5±9.3 | 82.7±1.4 | 31.1±0.8 | 51.6±1.0 |
| 15 | 541.6±5.1 | 187.9±1.0 | 368.5±1.9 | 90.1±0.6 | 34.8±0.5 | 55.3±0.3 |
| 16 | 508.8±6.1 | 188.6±2.3 | 360.0±4.4 | 87.4±0.9 | 29.3±0.5 | 58.2±0.7 |
| 17 | 527.9±10.5 | 189.3±1.4 | 372.0±2.7 | 93.5±0.7 | 34.4±0.6 | 59.1±0.4 |
| 18 | 438.5±4.6 | 157.7±4.1 | 359.6±9.4 | 90.6±1.1 | 31.3±1.2 | 59.3±1.6 |
| 19 | 462.2±3.7 | 180.4±2.6 | 350.0±5.0 | 94.0±0.7 | 32.7±0.8 | 61.3±0.9 |
| 20 | 582.7±2.9 | 160.6±4.8 | 350.2±10.4 | 80.3±0.2 | 35.4±1.1 | 44.9±1.3 |
| 21 | 546.9±1.4 | 170.9±0.5 | 293.1±0.8 | 80.9±0.1 | 36.0±0.1 | 45.0±0.1 |
| 22 | 505.4±2.5 | 187.0±2.0 | 314.1±3.3 | 80.9±0.3 | 36.3±0.2 | 44.6±0.5 |
| 23 | 684.4±3.9 | 236.8±3.1 | 440.9±5.8 | 91.5±0.7 | 37.6±0.2 | 53.9±0.7 |
| 24 | 627.0±13.9 | 145.0±2.6 | 307.3±5.5 | 60.7±0.7 | 29.6±0.2 | 31.1±0.6 |
| 25 | 598.9±7.8 | 175.1±3.0 | 401.2±6.8 | 82.7±0.6 | 33.7±0.6 | 48.9±0.8 |

**Table S5.** Total P, protein-associated P (mg/10 g protein), non-sedimentable (200 × *g*) P as a percentage of total P, 10 kDa-permeable P as a percentage of total P, and protein-associated P as a percentage of total P for formulae dispersions (samples 1-25) prepared by reconstitution of 12 g powder in 100 g water.

| Sample | Total P (mg/kg) | Protein-associated P (mg/10 g protein) | Non-sedimentable P (% of total P) | 10 kDa-permeable P (% of total P) | Protein-associated P (% of total P) |
| --- | --- | --- | --- | --- | --- |
| 1 | 488.3±7.4 | 136.1±3.2 | 96.7±1.1 | 50.4±0.8 | 46.2±1.1 |
| 2 | 488.7±10.3 | 141.9±0.9 | 92.3±0.3 | 43.5±1.1 | 48.8±0.3 |
| 3 | 455.6±19.9 | 179.1±5.9 | 92.3±1.7 | 40.0±0.7 | 52.3±1.7 |
| 4 | 708.9±7.7 | 151.7±5.5 | 75.7±1.3 | 40.0±0.3 | 35.7±1.3 |
| 5 | 378.9±12.1 | 119.2±10.9 | 98.8±4.6 | 49.2±2.4 | 49.6±4.6 |
| 6 | 491.3±11.6 | 177.2±11.0 | 98.7±3.3 | 45.7±0.6 | 52.9±3.3 |
| 7 | 460.4±4.7 | 198.5±10.6 | 93.3±2.9 | 39.3±0.5 | 53.9±2.9 |
| 8 | 477.5±7.6 | 147.4±5.0 | 82.5±1.3 | 44.7±0.9 | 37.8±1.3 |
| 9 | 395.8±4.7 | 185.1±1.9 | 92.1±0.6 | 34.2±1.6 | 57.9±0.6 |
| 10 | 424.9±14.7 | 175.1±12.2 | 97.7±3.5 | 47.1±1.8 | 50.6±3.5 |
| 11 | 518.5±10.0 | 156.0±7.4 | 97.1±2.3 | 47.5±2.6 | 49.5±2.3 |
| 12 | 479.8±9.4 | 116.4±6.7 | 91.8±2.0 | 57.5±3.6 | 34.2±2.0 |
| 13 | 463.1±11.4 | 130.6±5.7 | 73.5±1.7 | 34.4±2.7 | 39.1±1.7 |
| 14 | 438.3±4.4 | 124.3±7.8 | 85.4±2.8 | 40.1±1.1 | 45.3±2.8 |
| 15 | 546.8±20.6 | 157.3±10.3 | 94.8±3.0 | 48.9±5.3 | 45.9±3.0 |
| 16 | 496.7±16.2 | 158.9±12.2 | 97.6±3.9 | 47.4±1.6 | 50.2±3.9 |
| 17 | 522.4±11.6 | 126.6±8.7 | 93.5±2.8 | 53.6±2.1 | 39.9±2.8 |
| 18 | 441.8±10.6 | 122.3±2.2 | 97.7±0.8 | 52.1±2.4 | 45.6±0.8 |
| 19 | 455.3±11.0 | 130.1±4.2 | 98.8±1.4 | 53.9±3.9 | 44.9±1.4 |
| 20 | 556.5±18.2 | 121.9±9.5 | 97.8±2.8 | 62.1±2.7 | 35.7±2.8 |
| 21 | 433.6±7.1 | 111.4±8.4 | 94.6±2.8 | 57.7±2.4 | 36.9±2.8 |
| 22 | 437.5±15.5 | 109.3±14.9 | 82.6±4.1 | 52.5±0.7 | 30.1±4.1 |
| 23 | 493.7±13.7 | 121.1±0.7 | 93.2±0.2 | 55.0±2.7 | 38.2±0.2 |
| 24 | 472.8±17.9 | 96.0±3.8 | 70.2±1.1 | 42.8±0.2 | 27.4±1.1 |
| 25 | 486.4±7.2 | 102.6±3.3 | 91.4±1.2 | 56.1±0.7 | 35.3±1.2 |

**Table S6.** Total Mg, non-sedimentable (200 × *g*) Mg as a percentage of total Mg, 10 kDa-permeable Mg as a percentage of total Mg, protein-associated Mg (mg/10 g protein), and citrate content for formulae dispersions (samples 1-25) prepared by reconstitution of 12 g powder in 100 g water.

| Sample | Total Mg  (mg/kg) | Non-sedimentable Mg (% of total Mg) | 10 kDa-permeable Mg (% of total Mg) | Protein-associated Mg (mg/10 g protein) | Citrate content (g/kg) |
| --- | --- | --- | --- | --- | --- |
| 1 | 64.4±0.8 | 96.0±2.3 | 62.1±1.6 | 13.2±0.9 | 1.00±0.01 |
| 2 | 55.4±0.9 | 100.4±1.2 | 63.6±0.7 | 12.1±0.4 | 0.94±0.01 |
| 3 | 67.4±1.0 | 98.5±1.0 | 71.6±1.3 | 13.6±0.5 | 1.02±0.02 |
| 4 | 45.0±0.7 | 98.4±1.4 | 60.7±2.4 | 10.2±0.4 | 0.80±0.02 |
| 5 | 61.8±1.7 | 97.4±0.9 | 66.8±0.7 | 12.0±0.3 | 0.92±0.06 |
| 6 | 59.9±1.2 | 98.7±1.6 | 66.5±1.0 | 13.1±0.6 | 0.91±0.02 |
| 7 | 64.7±1.1 | 98.6±1.6 | 65.5±2.2 | 17.1±0.8 | 0.61±0.01 |
| 8 | 63.6±1.6 | 97.8±1.6 | 63.7±1.0 | 17.7±0.8 | 0.88±0.01 |
| 9 | 48.6±1.8 | 95.5±0.3 | 77.1±0.2 | 7.2±0.1 | 0.70±0.01 |
| 10 | 62.9±0.7 | 97.4±1.1 | 68.2±2.3 | 15.0±0.6 | 0.59±0.02 |
| 11 | 74.7±0.3 | 98.8±1.1 | 65.7±1.9 | 15.0±0.5 | 1.12±0.02 |
| 12 | 83.3±1.1 | 93.9±1.8 | 65.5±3.3 | 16.8±1.1 | 1.50±0.01 |
| 13 | 59.0±1.3 | 94.3±1.1 | 63.5±0.3 | 13.1±0.5 | 0.97±0.01 |
| 14 | 63.7±1.0 | 96.4±0.7 | 60.8±2.4 | 14.2±0.3 | 0.72±0.01 |
| 15 | 59.5±2.6 | 99.1±0.8 | 64.9±2.3 | 12.8±0.3 | 0.94±0.05 |
| 16 | 69.7±2.4 | 98.3±1.1 | 65.2±2.4 | 14.7±0.5 | 0.72±0.02 |
| 17 | 50.7±1.0 | 95.7±2.1 | 63.5±2.3 | 9.9±0.7 | 0.82±0.01 |
| 18 | 68.8±1.5 | 97.3±0.8 | 60.9±1.2 | 15.2±0.3 | 0.68±0.00 |
| 19 | 64.0±0.5 | 100.3±1.9 | 65.1±1.9 | 14.4±0.8 | 0.73±0.01 |
| 20 | 62.2±1.7 | 99.3±1.3 | 62.1±1.8 | 14.2±0.5 | 1.02±0.04 |
| 21 | 43.7±0.3 | 96.8±1.2 | 65.1±1.9 | 9.6±0.4 | 1.03±0.01 |
| 22 | 50.0±1.4 | 98.7±0.7 | 63.5±2.6 | 14.6±0.3 | 1.26±0.01 |
| 23 | 61.2±2.2 | 98.1±1.3 | 66.5±0.9 | 12.4±0.5 | 2.13±0.11 |
| 24 | 75.7±0.4 | 96.2±1.4 | 65.3±1.3 | 17.4±0.8 | 1.03±0.00 |
| 25 | 62.5±0.3 | 98.4±0.7 | 60.4±1.0 | 14.2±0.3 | 0.89±0.02 |

**Table S7.** Total K, total Na, non-sedimentable (200 × *g*) K as a percentage of total K, 10 kDa-permeable K as a percentage of total K, non-sedimentable (200 × *g*) Na as a percentage of total Na, and 10 kDa-permeable Na as a percentage of total Na for formulae dispersions (samples 1-25) prepared by reconstitution of 12 g powder in 100 g water.

| Sample | Total K  (mg/kg) | Total Na  (mg/kg) | Non-sedimentable K (% of total K) | 10 kDa-permeable K (% of total K) | Non-sedimentable Na (% of total Na) | 10 kDa-permeable Na (% of total Na) |
| --- | --- | --- | --- | --- | --- | --- |
| 1 | 763.9±3.3 | 225.3±5.4 | 98.2±0.9 | 96.7±1.0 | 98.8±2.2 | 94.0±0.9 |
| 2 | 684.3±2.8 | 210.5±1.8 | 101.6±0.7 | 92.4±0.3 | 98.5±1.4 | 95.4±0.3 |
| 3 | 577.0±6.0 | 270.0±3.2 | 97.5±0.7 | 98.1±0.5 | 96.4±1.8 | 96.2±0.9 |
| 4 | 652.3±1.7 | 143.7±3.0 | 97.6±0.2 | 93.0±0.7 | 97.7±0.6 | 93.0±0.7 |
| 5 | 648.2±17.6 | 248.7±5.2 | 100.0±0.8 | 96.8±0.7 | 99.3±1.1 | 95.5±0.6 |
| 6 | 785.5±5.3 | 263.3±1.7 | 97.8±0.9 | 95.4±0.2 | 100.3±1.3 | 93.4±0.2 |
| 7 | 702.1±12.3 | 225.0±4.7 | 99.6±0.5 | 98.0±0.4 | 100.1±1.0 | 95.0±0.3 |
| 8 | 765.2±2.9 | 216.7±4.9 | 96.7±0.2 | 96.9±0.6 | 99.2±3.9 | 92.4±1.4 |
| 9 | 654.8±17.9 | 197.3±6.0 | 99.3±1.1 | 98.1±0.2 | 100.1±1.2 | 96.5±1.2 |
| 10 | 694.0±3.4 | 289.9±5.5 | 97.2±0.8 | 96.3±0.8 | 98.9±2.3 | 95.3±0.6 |
| 11 | 887.2±5.9 | 283.7±0.8 | 98.3±2.3 | 98.6±0.3 | 97.3±1.6 | 95.9±0.7 |
| 12 | 724.0±4.6 | 257.7±3.7 | 98.7±0.3 | 99.2±0.4 | 98.2±1.7 | 97.0±1.3 |
| 13 | 760.6±5.4 | 218.5±3.8 | 99.2±0.4 | 94.0±0.9 | 95.9±0.6 | 93.2±1.4 |
| 14 | 616.2±1.6 | 237.1±3.8 | 96.7±0.7 | 94.1±1.3 | 98.1±0.3 | 90.6±1.0 |
| 15 | 772.1±10.1 | 207.5±5.8 | 97.9±0.8 | 90.5±0.3 | 98.0±0.9 | 96.4±0.3 |
| 16 | 786.7±3.0 | 219.1±4.8 | 97.1±0.3 | 90.1±0.0 | 97.3±0.5 | 90.5±1.8 |
| 17 | 716.1±1.8 | 203.9±2.2 | 95.0±2.4 | 89.9±1.2 | 97.3±0.4 | 90.9±0.2 |
| 18 | 758.7±9.6 | 204.2±3.7 | 96.7±1.0 | 89.2±0.6 | 98.4±1.3 | 93.9±1.0 |
| 19 | 654.3±4.4 | 209.3±1.4 | 99.3±1.9 | 96.0±0.5 | 99.2±3.2 | 96.8±1.1 |
| 20 | 869.2±6.9 | 272.3±2.3 | 97.0±1.5 | 96.8±0.1 | 99.1±1.4 | 98.7±0.8 |
| 21 | 915.9±0.7 | 193.7±1.1 | 97.4±0.7 | 95.1±0.8 | 99.6±0.7 | 96.0±1.5 |
| 22 | 760.2±10.5 | 207.9±1.3 | 98.0±0.2 | 95.7±1.2 | 96.8±0.4 | 95.3±1.1 |
| 23 | 852.8±6.7 | 308.2±7.2 | 97.5±0.7 | 96.0±0.9 | 97.6±0.5 | 92.7±1.3 |
| 24 | 889.5±6.5 | 237.1±0.7 | 99.6±0.2 | 95.5±0.7 | 98.0±0.6 | 94.4±0.5 |
| 25 | 749.2±1.8 | 256.4±1.1 | 99.2±1.4 | 96.5±0.5 | 99.9±1.0 | 95.3±1.7 |

**Table S8.** Furosine content, blocked lysine as a percentage of total lysine, reactive lysine content, and HMF content for formulae dispersions (samples 1-25) prepared by reconstitution of 12 g powder in 100 g water.

| Sample | Furosine (mg/100 g protein) | Blocked lysine (% of total lysine) | Reactive lysine (g/100 g protein) | HMF (mg/100 g protein) |
| --- | --- | --- | --- | --- |
| 1 | 562.6±0.7 | 12.2±0.1 | 7.3±0.1 | 3.77±0.05 |
| 2 | 616.5±0.5 | 13.9±0.1 | 6.9±0.1 | 3.96±0.20 |
| 3 | 729.3±2.2 | 17.3±0.2 | 6.3±0.1 | 6.51±0.08 |
| 4 | 621.5±1.1 | 13.9±0.4 | 6.9±0.2 | 5.18±0.08 |
| 5 | 692.6±4.7 | 15.9±0.7 | 6.6±0.3 | 4.72±0.07 |
| 6 | 743.4±2.8 | 15.4±0.1 | 7.3±0.1 | 5.22±0.11 |
| 7 | 390.8±0.9 | 8.9±0.2 | 7.2±0.1 | 3.82±0.06 |
| 8 | 494.6±3.5 | 11.0±0.2 | 7.2±0.1 | 4.43±0.08 |
| 9 | 528.3±2.4 | 11.5±0.1 | 7.3±0.1 | 4.89±0.03 |
| 10 | 514.3±2.8 | 11.4±0.4 | 7.2±0.3 | 4.84±0.04 |
| 11 | 666.0±3.0 | 14.4±0.7 | 7.1±0.4 | 4.78±0.06 |
| 12 | 503.8±3.0 | 10.4±0.1 | 7.8±0.1 | 4.29±0.02 |
| 13 | 605.9±3.1 | 12.9±0.2 | 7.4±0.1 | 7.01±0.10 |
| 14 | 552.5±2.8 | 10.3±0.2 | 8.7±0.2 | 3.82±0.06 |
| 15 | 713.7±2.2 | 14.0±0.2 | 7.9±0.1 | 5.30±0.01 |
| 16 | 699.0±8.0 | 13.3±0.2 | 8.2±0.2 | 5.46±0.06 |
| 17 | 746.8±1.3 | 14.7±0.3 | 7.8±0.2 | 5.50±0.03 |
| 18 | 877.2±1.3 | 17.9±0.6 | 7.2±0.3 | 6.26±0.06 |
| 19 | 791.5±2.4 | 16.4±0.4 | 7.3±0.2 | 6.31±0.06 |
| 20 | 829.3±6.8 | 15.9±0.4 | 7.9±0.2 | 5.30±0.02 |
| 21 | 327.2±3.3 | 7.4±0.1 | 7.3±0.1 | 3.00±0.03 |
| 22 | 661.9±3.7 | 13.8±0.4 | 7.4±0.2 | 6.36±0.05 |
| 23 | 546.8±0.8 | 11.1±0.1 | 7.9±0.1 | 4.34±0.10 |
| 24 | 602.9±1.2 | 12.7±0.4 | 7.4±0.2 | 5.69±0.06 |
| 25 | 898.5±2.0 | 19.2±0.2 | 6.8±0.1 | 5.18±0.04 |
